# Isomeric Differences in Nanoparticle’s Surface Chemistry Alter Macrophage Interactions In Vitro Due to Protein Corona

**DOI:** 10.1021/acsomega.5c03123

**Published:** 2025-09-11

**Authors:** Sridevi B. Conjeevaram, Amulya Kadaba, Isaac M. Adjei

**Affiliations:** Department of Biomedical Engineering, 2655 Texas A&M University, College Station, Texas 77843, United States

## Abstract

The interactions of nanoparticles (NPs) with macrophages after intravenous injection are critical in determining their biodistribution and pharmacokinetics. The formation of protein corona (PC) on the nanoparticles, often a result of NP-plasma protein interactions, influences macrophage response, thereby directly determining the NPs’ fate *in vivo*. While the effect of NPs’ surface chemistry on protein corona formation and macrophage interaction is extensively studied, the results are often confounded by the effects of surface charge and hydrophobicity. Therefore, to assess the impact of NPs’ surface chemistry alone on protein corona and NP-macrophage interactions, we developed gold (Au) NPs surface-modified with the isomeric amino acids leucine (Leu) and isoleucine (Iso Leu), which have similar isoelectric points and hydropathy indices. The synthesized NPs had similar sizes, zeta potentials, hydrophobicities, and morphology. Incubation of the NPs in plasma under flow conditions resulted in a protein corona (PC) that differed in amount and composition. Pathway analysis showed that these variations in the PC corresponded to the activation of different phagocytic and inflammatory pathways. These in silico analyses were confirmed in macrophages, which showed higher uptake of the Leu-AuNP-PC complex compared to Iso Leu-AuNP-PC and a corresponding increase in inflammatory cytokines. These studies demonstrate the sensitivity of the PC to subtle changes in the surface chemistry of NPs, which could have significant in vivo implications for the clinical application of NPs.

## Graphical abstract

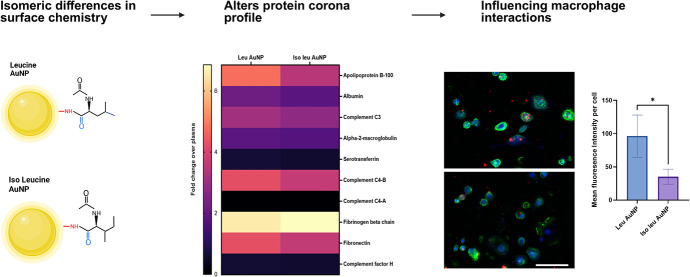

## Introduction

The application of nanoparticles (NPs) for disease diagnosis and drug delivery continues to increase in modern medicine.^1^ For most applications, the effectiveness of diagnosis or therapy depends on the delivery of NPs to the correct tissues. However, the rapid clearance of intravenously injected NPs by the mononuclear phagocytic system (MPS) remains a challenge for their application in drug delivery and imaging.^2^ Macrophages, particularly Kupffer cells in the liver, remove the injected nanoparticles from the circulation, reducing the nanoparticle’s half-life and influencing their biodistribution.^3^ Critical recognition mediated by macrophages and NP endocytosis is the protein corona (PC) that forms on NPs’ surfaces as they interact with biological fluids.^4^ Opsonins in the protein corona elicit an immunological response from the MPS, triggering phagocytosis and cytokine release to restore the body to homeostasis. This directly affects the pharmacokinetics and biodistribution of NPs *in vivo*, reducing their therapeutic efficacy.

The physicochemical properties of the NP and the incubation environment influence the composition of the PC. The NPs’ surface affects the protein corona composition through interfacial interactions between NPs and biological fluid. Surface properties of the NPs, such as surface charge, chemistry, and hydrophobicity, affect protein recruitment through hydrophobic forces,^5^ H-bonding, or electrostatic interactions,^6^ seeding a protein shell that grows to form the PC. Due to its essential role in determining the PC, modifying surface chemistry is an attractive approach for changing the biological behavior of NPs. However, surface chemistry is intertwined with surface charge and hydrophobicity, with a change in NP surface chemistry usually also resulting in a change in these other two physicochemical characteristics.^7,8^

Previous studies have highlighted the central role that the surface chemistry of an NP plays in determining NPs’ interactions with plasma proteins and macrophages.^9–11^ With increasing complexity in surface modifications of NPs to achieve different clinical goals, it has become necessary to identify how these changes, however subtle, could influence the PC and macrophage interaction. Unfortunately, the sensitivity of the PC to subtle changes in surface chemistry has been challenging to explore because of a lack of a framework that enables the evaluation of the effects of surface chemistry without the confounding effects of surface charge or hydrophobicity.

Amino acids are small organic molecules with amine and carboxylic functional groups and a unique side chain that ensures their use for chemical modification with relative ease. The wide variety of amino acids, with side chains that provide molecules with similar hydrophobicity and isoelectric points, allows investigation into how subtle surface chemistry changes impact macrophage interactions with NPs.^12^ The amino acids leucine and isoleucine are constitutional isomers^13^ that have similar isoelectric points (∼6.04) and hydropathy indices of 1.6 (leucine) and 1.7 (isoleucine)^13–15^ (Figure 1). The similarities in their isoelectric properties and hydropathy indices should ensure that the NPs they modify have similar physicochemical properties and differ only in the structural arrangement of the bonds. These similarities should ensure the evaluation of the effects of surface chemistry alone, without the influence of charge and hydrophobicity.

**Figure 1:**
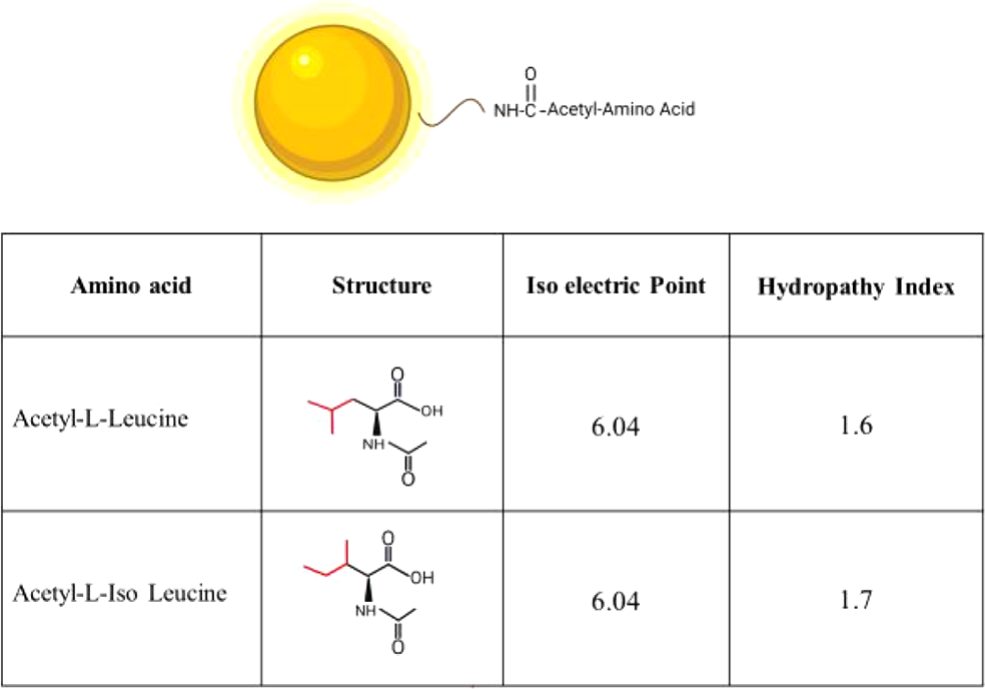
Nanoparticle design. Cationic gold nanoparticles were conjugated to acetyl-l-amino acids Leucine and Isoleucine via EDC-NHS chemistry, forming an amide bond. The table highlights the structure and isoelectric point of each amino acid.

In this study, amino acids were conjugated to the surface of gold NPs via EDC-NHS chemistry to evaluate the sensitivity of the protein corona formed on NPs to minute changes in surface chemistry and to address the effects on macrophage interactions (Figure 1). To facilitate the conjugation process and prevent unwanted binding, we used acetylated amino acids, i.e., acetyl-l-Leucine (Leu) and acetyl-L-Iso leucine (Iso Leu). The acetyl group shielded the amine terminals on the amino acids, preventing self-ligation while exposing the carboxylic group for conjugation. Although the amino acids are isomers, the resulting NP system had similar surface charge, hydrophobicity, size, and morphology. Using these AuNP systems, we show that the PC amount and composition are sensitive to isomeric surface chemistry differences. The differences in the PC composition affect the NPs’ uptake and toxicity to macrophages. Informatics analysis of the PC demonstrates that isomeric differences in surface chemistry influence the enrichment of proteins associated with phagocytosis.

## Results and Discussion

### Characterization of Gold Nanoparticles with Similar Size and Charge but Different Surface Chemistry

Cationic AuNPs have previously been synthesized by reducing gold salts with alkylamine surfactants.^16^ However, this synthesis is performed in organic solvents, such as toluene, requiring phase transfer into aqueous solutions. Alternatively, citrate-capped AuNPs are modified with cationic groups to produce cationic AuNPs.^17^ Here, gold salt was reduced with the polyelectrolyte poly­(allyl)­amine hydrochloride (PAH) in a one-step method that produces cationic AuNPs that are stable in aqueous solutions and have available amines for further modifications. The resulting particles were PEGylated using thiol-PEG-amine to improve the ionic stability. The synthesized particles, termed Bare AuNPs, had an average hydrodynamic diameter of 37 ± 5 nm (Figure 2A) and a core diameter of 23 ± 3 nm measured via transmission electron microscopy (TEM) (Figure 2B,C). Particles with different surface chemistries but similar surface charges were generated from the Bare AuNPs by conjugating the amino acids acetyl-l-leucine and acetyl-l-isoleucine to the free amines from the poly­(allyl)­amine hydrochloride and thiol-PEG-amine. To prevent the self-reaction of the amino acids, acetylated amino acids (the acetyl group blocks the −NH2 terminal) were used for the surface modifications. While the amino acid-conjugated AuNPs had similar TEM diameters as the Bare AuNPs, their hydrodynamic sizes were significantly reduced, with leucine AuNP (Leu AuNP) and isoleucine AuNP (Iso Leu AuNP) having average hydrodynamic sizes of 23 and 25 nm, respectively (Figure 2A). The hydration shell that contributes to the hydrodynamic diameter of NPs is influenced by the NPs’ surface hydrophilicity, which is decreased by the addition of the hydrophobic amino acids.^18^ The hydrophobicity of the NPs after amino acid conjugation was confirmed by their partitioning into octanol, measured as their log *p* value. Log *p* measures solute partitioning into the aqueous phase when placed in an oil/water mixture. A negative log *p* value indicates that the molecules under investigation are hydrophilic. Bare AuNPs had a log *p* value of −1.3. In contrast, Leu and Iso Leu had values of −0.33 and −0.37, respectively, confirming the decreased hydrophilicity and hence the reduced size of hydration shells and hydrodynamic diameter.

**Figure 2:**
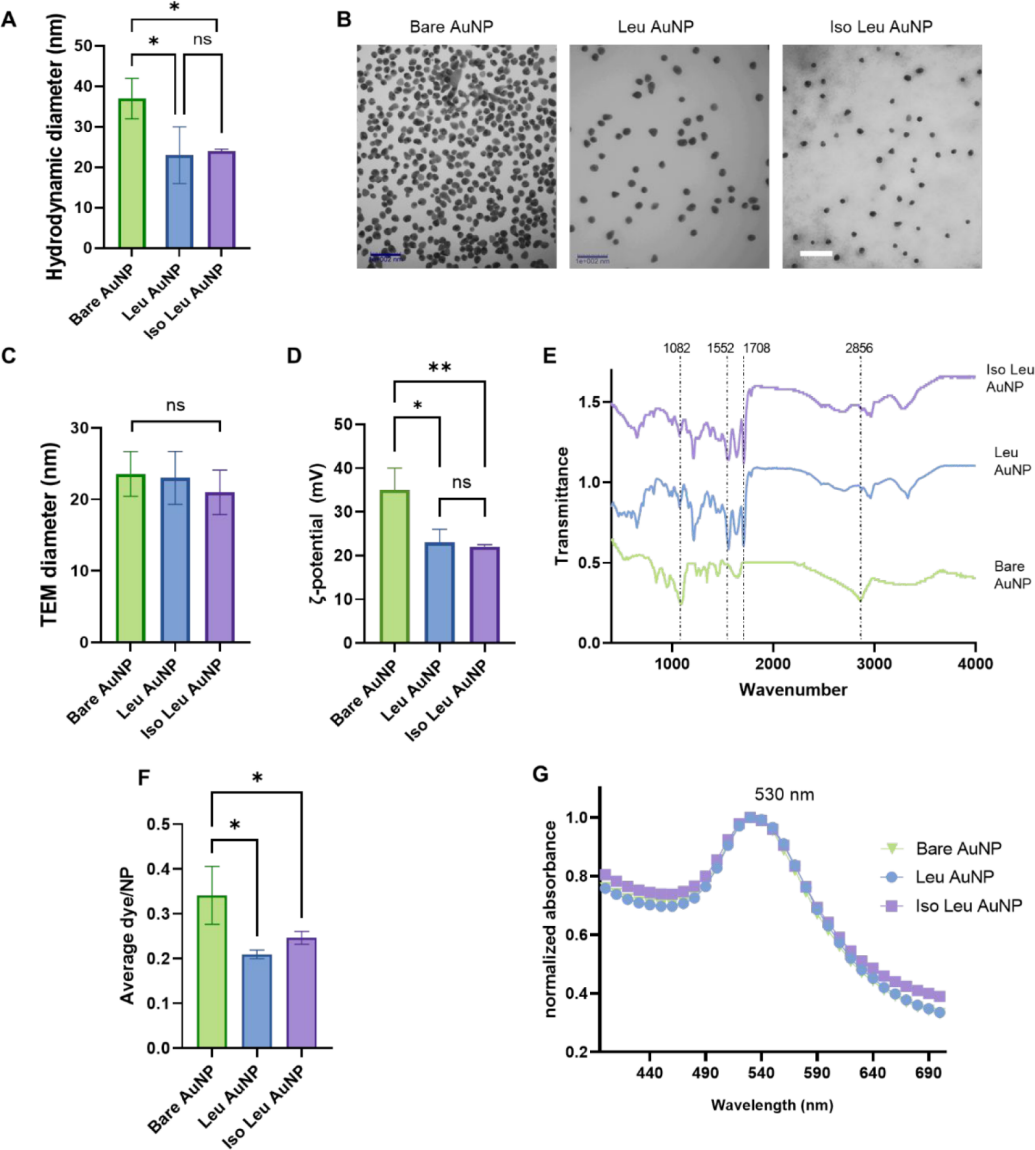
Nanoparticle characterization and confirmation of conjugation: (A) Hydrodynamic diameter of NPs. (B) Transmission electron microscope micrograph (TEM) and (C) TEM size distribution of the different Au NPs. (D) Zeta potential measurements of NPs. (E) FT-IR spectra of bare and amino acid conjugated NPs. (F) Fluorescence reading of dye conjugated NPs, (G) UV–visible spectroscopy analysis of Bare, Leu and Iso-leu conjugated AuNPs. Conjugation of amino acids to the nanoparticles reduced the AuNPs’ hydrodynamic radii and zeta potential values, whereas the NPs’ morphology, true size, and physical properties remains unaltered, confirming the conjugation of NPs. Dye conjugation and FTIR peaks support the successful conjugation of amino acids Leu and Iso Leu to the NP surface via a reduction in available amines and the presence of amide bonds. (One-way ANOVA followed by post hoc Tukey’s test, *n* = 3, *p < 0.05, **p < 0.01).

The free polyamines from poly (allyl)­amine and the amine-capped PEG resulted in Bare AuNPs with a zeta potential of +36 ± 5 mV, which decreased after amino acid conjugation. The decreased free amines and presence of anionic acetyl groups reduced the zeta potential to 23 ± 3 and 22 ± 0.5 mV for Leu AuNP and Iso Leu AuNP, respectively, and the difference was not statistically different (Figure 2D).

The conjugation of the amino acids to the NPs was confirmed by Fourier-transform infrared spectroscopy (FTIR; Figure 2E). The conjugation of the carboxylic groups of the amino acids to the amines on AuNPs via EDC-NHS chemistry results in a covalent amide bond, which was identified by FTIR analysis. The stretching of the C=O bond in the amide linkage is observed at 1708 cm^–1^, while the N–H bending in the amide bond is measured at 1552 cm^–1^ in the FTIR plots of the amino acid-conjugated AuNPs. These unique amide bond-related bands are absent in the FTIR spectrum for the Bare AuNPs.^19^ The effect of the new chemical groups on the spectrum shifts was also observed after the conjugation of the amino acids. The alcohol stretches from PEG, present at 1082 cm^–1^ on Bare AuNPs, shift to 1207 cm^–1^ on the amino acid-conjugated NPs. Similarly, the C–H stretching from PEG and PAH shifts from 2856 cm^–1^ in the Bare AuNPs to 2943 cm^–1^ in the amino acid-conjugated AuNPs. The peak at 3286 cm^–1^ corresponds to the methyl (CH_3_) group observed on amino acids and is absent on Bare AuNPs.^20^ The strong N–H stretch observed in Leu and Iso Leu AuNPs, and the broad N–H stretch on Bare AuNP at 3300 cm^–1^, suggest the presence of available amines on the surface. This observation corresponds to the positive zeta potential observed on them.^21^

The degree of amino acid functionalization of the AuNP surface was determined via a dye conjugation technique. This method measures the available free amines on the AuNP surface by assessing their reactivity to an NHS-ester dye.^22^ The amount of dye conjugated to Leu-AuNP and Iso Leu was significantly lower than that on the Bare AuNPs (Figure 2F). There was no difference in the amount of dye conjugated to two amino acid-conjugated AuNPs.

Metal NPs often exhibit strong localized surface plasmon resonance (LSPR), and the wavelength corresponding to the maximum LSPR activity is denoted as LSPR λ_max_. The LSPR often dictates NPs’ properties, including optical and electrical characteristics.^23^ Gold NPs exhibit plasmon resonance in the UV–visible range of the electromagnetic spectrum, and its λ_max_ changes with the NPs’ physical properties such as shape, size, and surface modifications.^24^ Here, we observe that amino acid conjugation did not affect the absorption characteristics of the AuNPs, with all particle systems exhibiting the characteristic spherical AuNP absorbance spectrum with λ_max_ at 530 nm (Figure 2G).

### Protein Corona on NPs Alters the NPs’ Physicochemical Properties

The interactions of the NPs with plasma were performed under dynamic flow conditions to model physiological conditions (Figure 3A). Since hydrophobicity and charge of NPs correlate to the number of proteins that adsorb to them, it was expected that Leu AuNPs and Iso Leu AuNPs would have a similar quantity of bound proteins. However, Iso Leu AuNPs had 50% more adsorbed proteins than Leu AuNPs (Figure 3B).

**Figure 3:**
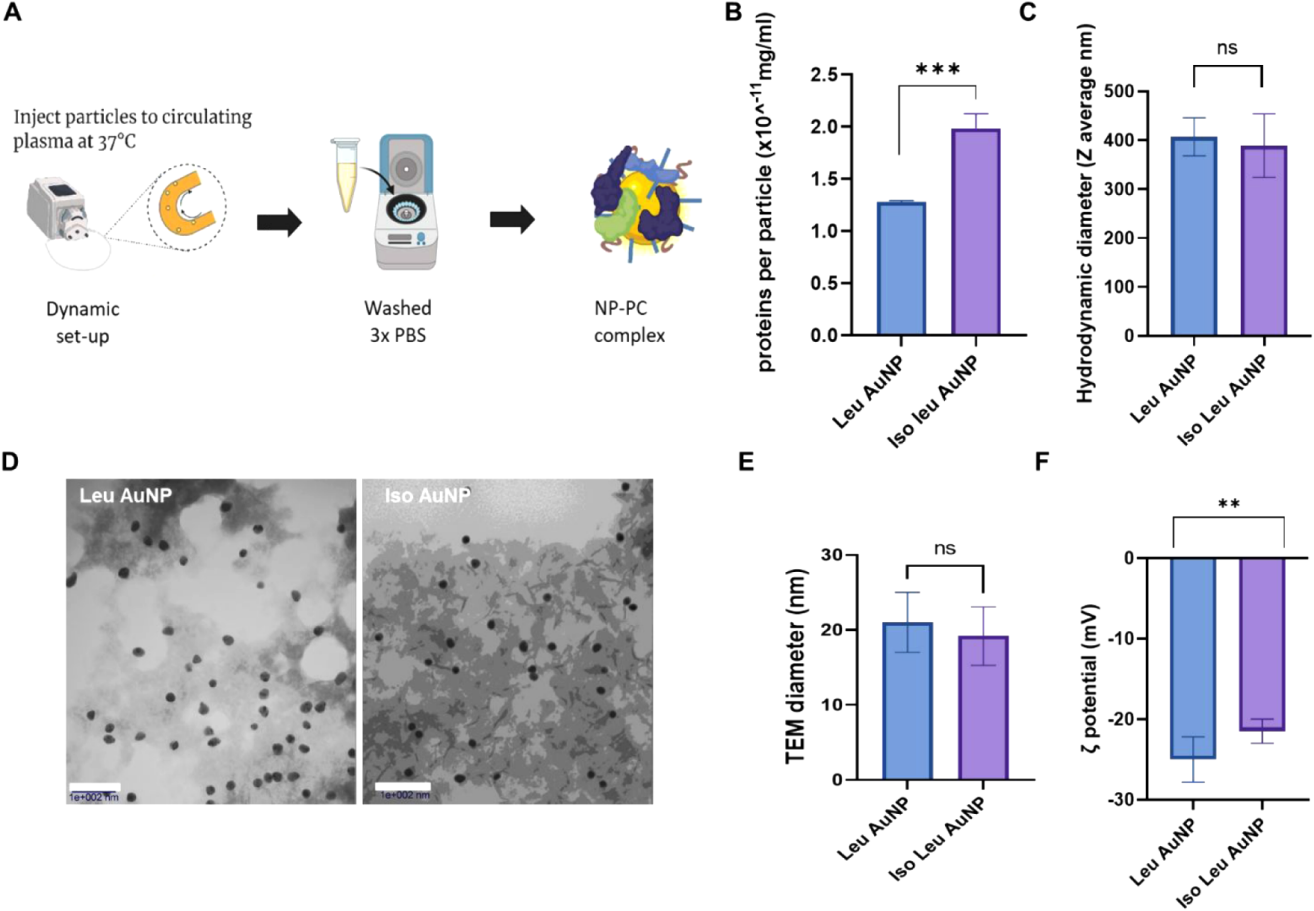
NP characterization after the formation of a protein corona. (A) Schematic of NP interaction with plasma proteins in a dynamic setup, with plasma proteins circulating in tubing via a peristaltic pump. (B) Hydrodynamic diameter of amino acid-conjugated nanoparticles post incubation in plasma. (C) Protein concentration in the protein corona formed on AuNPs, measured by micro-BCA assay. (D) TEM image and (E) Corresponding size distribution of nanoparticles after incubation in circulating plasma proteins. (F) Zeta potential analysis of NPs after plasma protein interaction. The protein corona altered the physicochemical properties of the nanoparticles, increasing the NPs’ hydrodynamic radii and decreasing their zeta potential values. (*t*-test, *n* = 3, **p < 0.01, ***p < 0.001,).

The interaction of the NPs with plasma changed their physicochemical characteristics. While the pristine amino acid-modified NPs had hydrodynamic diameters of 20 – 30 nm, their hydrodynamic sizes (Z-average) increased at least 10x after interaction with plasma (Figures 3C and S1). Protein adsorption to NPs can result in their aggregation and lead to increased size.^25^ Generally, the higher the amount of adsorbed proteins, the greater the tendency to aggregate. The DLS number, intensity, and volume average distribution profiles further confirm post-corona aggregation on both Leu- and Iso Leu–AuNPs, as evident from multiple peaks at higher diameters (Figure S1). While Pristine Leu-AuNPs exhibit a bimodal number- and intensity-weighted profile centered at ∼20 nm, after plasma exposure, both distributions shift to larger diameters with a new secondary peak emerging around ∼1 μm in the intensity mode—a direct indication of small NP clusters. Pristine Iso Leu-AuNPs follow a similar trend: the number-weighted peak moves beyond 20 nm, and the intensity-weighted profile develops three peaks between 100 nm and 10 μm (Figure S2). Because intensity weighting overemphasizes large scatterers, the secondary peak at high diameters confirms a minor population of aggregates, while the two main peaks reflect NPs with protein corona distributed over two diameters, i.e., at 100 nm and ∼1000 nm. Inorganic nanoparticles tend to aggregate in protein-rich environments, especially at higher protein concentrations, as evident by bimodal peaks in intensity-weighted distributions.^26^ Bare gold nanoparticles (AuNPs) typically undergo destabilization and aggregation in protein-rich solutions prior to the formation of the protein corona, indicating the need for surface modifications to maintain NP stability.^27^ While Bare AuNPs typically recruit a thick protein corona, surface modification techniques such as PEGylation reduce the hydrodynamic diameter and help preserve the intended biological fate.^28^ In this study, Bare AuNPs underwent PEGylation for increased colloidal stability, which were further modified with amino acids Leucine and Iso leucine, thereby potentially altering the thickness of individual protein corona while introducing aggregation in the system.

Immediately after plasma exposure, the ζ-potential of both Leu- and Iso Leu AuNPs drops from +34 to −25 mV within 10 min, confirming rapid adsorption of anionic plasma proteins (Figure 3F). Over the next 50 min, the potential drifts toward neutrality, stabilizing to ∼−8 mV by 60 min and remaining constant out to 120 min (Figure S3). This profile is characteristic of the Vroman effect: fast, reversible binding of abundant proteins followed by slower exchange with higher-affinity species until the corona equilibrates. The identical trajectories for the two AuNPs indicated that surface chemistry did not affect long-term colloidal stability once the protein corona was established.

However, the TEM images of the NPs after incubation in plasma (Figure 3D,E) did not show any NP aggregates, instead showing distinct individual NPs dispersed on the TEM grid with an average core size similar to that of the pristine NPs. Although the core particle sizes remained the same as the pristine NPs, the TEM images showed that the NPs were embedded in protein matrices, which may account for the larger hydrodynamic size observed using dynamic light scattering.

The proteins associated with the NPs resulted in zeta potential reversal from cationic in the pristine AuNPs to negative (Figure 3F). Plasma proteins are mostly anionic, and their interaction with the cationic NPs could result in the observed charge reversal.^29^ Interestingly, the zeta potential of the protein corona-NPs (NP-PC) complex did not correspond to the quantity of the associated proteins. This initial observation may confirm differences in the types of proteins associated with the NPs. Proteins have different isoelectric points, and the sum of the charges of the proteins associated with Iso Leu AuNPs may make them less anionic than Leu AuNPs.

### Protein Corona Composition is Sensitive to Isomeric Changes in Surface Chemistry

The composition of the protein corona determines how it affects the *in vivo* fate of the NP. The presence of opsonins could enhance their clearance by the MPS, while dysopsonins inhibit the clearance and increase the half-life of NPs.^30,31^ As such, the proteins associated with the NPs and their relative abundance were analyzed by LC-MS/MS. For accurate PC characterization, a protein must be identified in at least 2 out of 3 trials to be included in the analysis.^32^ While over 1000 proteins are present in plasma, 165 and 184 proteins were identified in the PC of Leu AuNP and Iso Leu AuNP, respectively (Table S1). Of the proteins identified in the corona, 110 were shared between the Leu AuNP and Iso Leu AuNP (Figure 4A).

**Figure 4:**
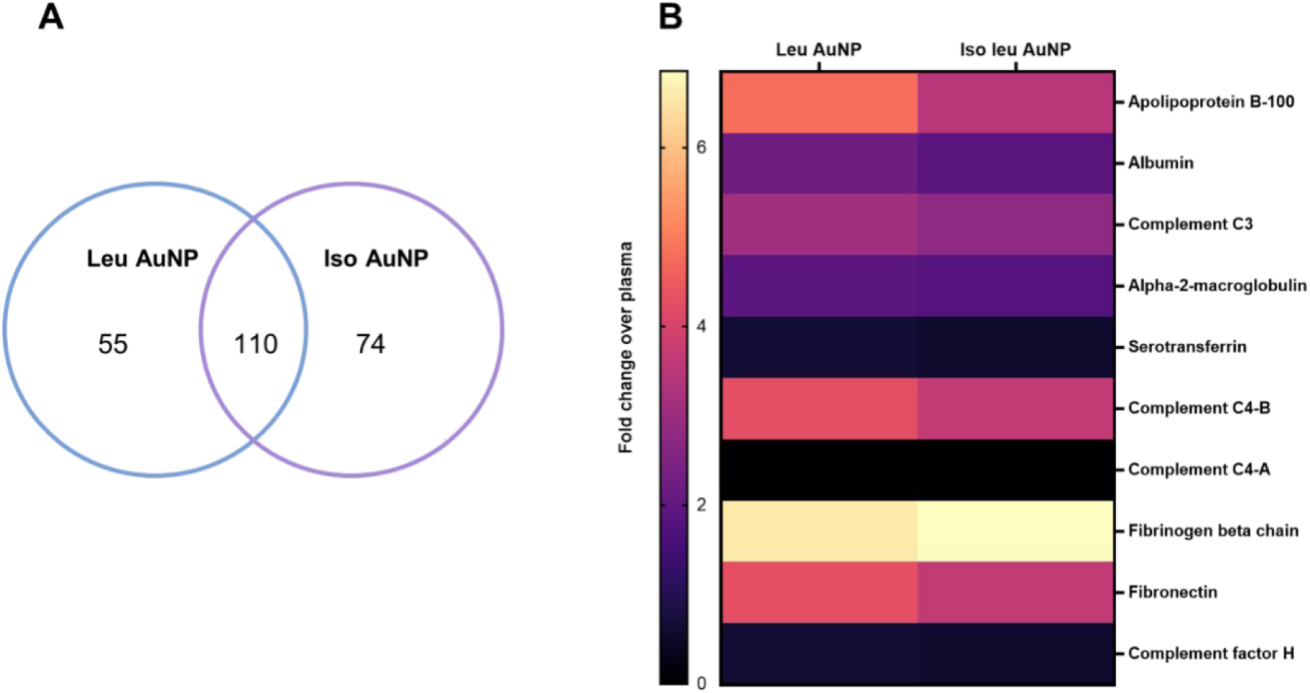
LC-MS/MS analysis of the protein corona. (A) Venn diagram showing the number of common and different proteins identified in the protein corona of Leu and Iso Leu AuNPs. (B) Heatmap showing the enrichment of the top 10 most abundant plasma proteins on Leu AuNP and Iso Leu AuNP (average log fold change).

We next assessed if the abundance of proteins, reported as average log fold changes over plasma, in the protein corona was dependent on their abundance in plasma. Most of the top plasma proteins were present on the protein corona of both NPs, except for complement C4A, which was not present on either NP (Figure 4B and Table S2). While most of the proteins were enriched on both AuNPs, the degree of adsorption was different, highlighting how the isomeric differences in their surface chemistries could affect the PC composition.

Interestingly, the most abundant plasma proteins adsorbed to the NPs contribute toward defending the body from foreign invasion (except albumin, which helps maintain the osmotic pressure). The presence of such proteins predominantly works to remove NPs from circulation, posing a significant barrier to successfully translating NPs into therapeutics and diagnostics.

To further investigate the role of surface chemistry on NP biodistribution, the proteins identified from LC-MS/MS were categorized according to their biological functions: acute phase reactants, immunoglobulins, complements, coagulation, and lipoproteins. Previous studies have shown that an amine end group on NPs resulted in a rich abundance of apolipoproteins (∼5-fold), followed by acute phase, coagulation, and complement proteins, while immunoglobulins were de-enriched in the corona.^33^

Here, we report a ∼1.35-fold enrichment (±0.4 and ± 0.1; CI: 0.2–2.4 and 1–1.3 for Leu AuNP PC and Iso Leu AuNP PC, respectively) of proteins associated with acute phase reactions on both NPs relative to plasma, while the abundance of complement system and coagulation-associated proteins increased ∼3-fold (Leu AuNP PC: 3.04 ± 0.5; CI: 1.8–4.3 and 2.6 ± 0.3; CI: 1.0–4.2, and Iso Leu: 2.8 ± 0.9; CI: 2.3–3.5 and 2.9 ± 0.2; CI: 0.45–5.3 respectively) on the NPs relative to plasma, an observation remarkably similar to those reported by Saha et al. for cationic AuNPs.^34^ Lipoproteins were enriched more than 4-fold (Leu AuNP PC: 5 ± 01.5; CI: 4.6–5.3, and Iso Leu AuNP PC: 3.6 ± 1.34; CI: 0.3–7) on the NPs relative to plasma, likely due to the hydrophobicity of their surface chemistries.^35^ Interestingly, a 0.6-fold ± 0.1 (CI: 0.15–1.09) decrease in the abundance of immunoglobulins relative to plasma was observed on Iso Leu AuNP only (Figures 5 and S1).

**Figure 5:**
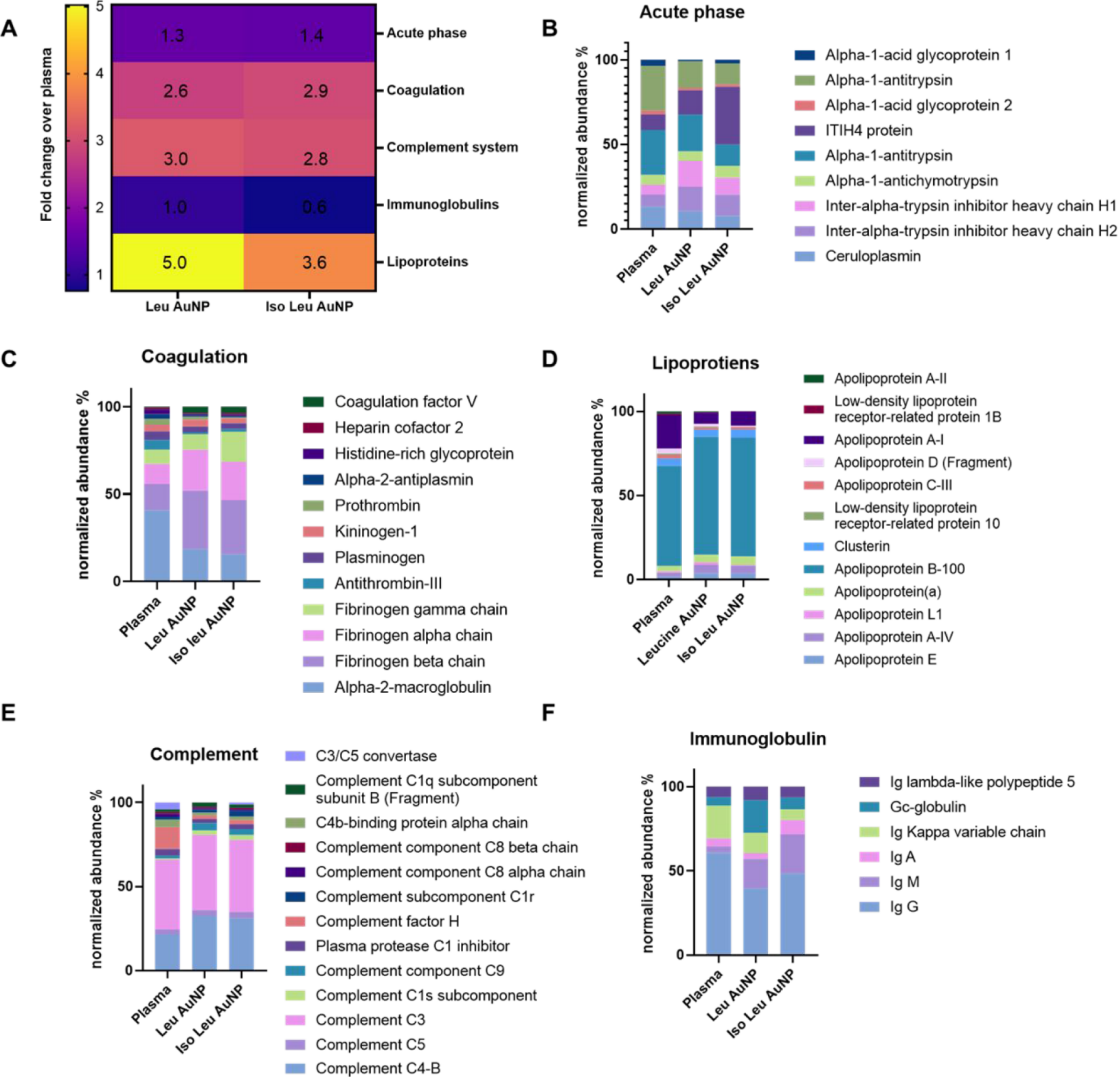
(A) Heatmap showing the average distribution of proteins classified according to their function. Individual proteins in the corona contributing to the biological processes, viz, (B) acute phase proteins, (C) coagulation, (D) lipoproteins, (E) complements, and (F) Immunoglobulins. While acute phase, coagulation, lipoproteins, and complement proteins were enriched on the NP surface, immunoglobulins on Iso Leu AuNP showed a reduction.

These findings are in close agreement with current reports of protein enrichment on amine-terminated nanoparticles. The most enriched proteins in the coronae are the lipoproteins, while plasma is mostly enriched in albumin and complement proteins. Such lipoprotein enrichment has been previously reported to be directly dependent on the location of the −NH_2_ end group on the nanoparticle.^36^ Moreover, these interactions are proposed to be driven by electrostatic interactions between the charged lipoproteins and the nanoparticle surface, with possible aggregation over time.^37^ The enrichment of certain protein groups on NPs relative to their abundance in plasma is a common phenomenon and contributes to differing biological effects. Such enrichments have also been used in various applications, such as targeted therapy and diagnostics, by modifying the surface chemistry of NPs to trap specific analytes, exponentially increasing the sensitivity of assays.^38^

Since the two NPs showed similar average increases or decreases in enrichment of the different protein classes, we analyzed if the individual protein components and their abundances were similar. Acute phase proteins mediate the innate immune response and are critical to the body’s first-line defense against foreign material, increasing or decreasing in response to inflammation.^39^ While the overall abundance of acute phase proteins is similar between the two surface-modified NPs, the type and abundance of the proteins differ significantly (Figure 5B and Table S3). Alpha-1-acid glycoprotein, a lipophilic affinity protein and the most abundant acute phase protein identified on any of the NPs, is enriched ∼2 ± 1.6 on Iso Leu AuNP than the Leu AuNP.

These phenomena show that hydrophobic indices alone do not drive protein enrichment in the coronae. Furthermore, Bare AuNPs have been reported to show an enhanced preference for alpha-1-antitrypsin, a protein whose role is correlated with anti-inflammatory functions, specifically in the lungs.^40,41^ While the Leu AuNPs show equal abundance of Alpha-1-Antitrypsin in the plasma, a 10-fold decrease in enrichment is observed on Iso Leu AuNP, indicating that the surface chemistry has the potential to alter NPs’ affinity to even high-affinity proteins—a plausible reason could be the individual properties of the Iso leucine amino acid.

Coagulation-related proteins were enriched ∼2.8 ± 0.7 (CI 1.93–3.52) folds on both the nanoparticles; individual proteins contributing to the subfamily showed remarkable differences in their abundance (Figure 5C and Table S3). For example, fibrinogen emerged as the most abundant coagulation protein in the nanoparticle (NP) corona. The alpha and beta chains were present at comparable levels on both Leu and Iso Leu AuNPs; however, the gamma chain showed a marked difference in abundance between the two surfaces. All three chains were cysteine-rich and could adsorb directly to AuNPs, accounting for their overall enrichment. While bulk fibrinogen binding to AuNPs is often described as surface-charge–independent, our data reveal that the individual subunits are sensitive to subtle variations in surface chemistry,^42,43^ Importantly, the gamma chain harbors the thrombin-binding motif that initiates downstream fibrin clot formation; its differential recruitment therefore highlights how small compositional shifts in the corona can translate into outsized biological effects.^44^ In general, Bare AuNPs have an affinity toward fibrinogen proteins, which causes irreversible aggregation of AuNPs, even in the presence of other proteins, as shown by Kharazian et al.^45^ The increased affinity to the gold NP surface leads to a conformational change in fibrinogen’s inherent structure, resulting in an increase in inflammatory cytokine release and downstream immune responses.^46^

Interestingly, the two surfaces skew the corona toward opposite ends of the coagulation cascade. Leu AuNPs preferentially recruit procoagulant factors—most notably prothrombin (2.3 ± 0.3-fold; CI: 0–5.53) and plasminogen (1.3 ± 0.8-fold; CI: 0–3.2). By contrast, Iso Leu AuNPs are enriched in anticoagulant regulators, including heparin cofactor II (1.4 ± 1.2-fold; CI: 0–4.4) and antithrombin III (1.3 ± 1.6-fold; CI: 0–5.4). Although all of these proteins fall under the broad “coagulation” category, their opposing functions illustrate how subtle differences in surface chemistry can shift the functional balance of the corona and, consequently, the fate of the nanoparticles.

Lipoproteins are plasma proteins that aid in lipid transport and immune clearance.^47^ Although Leu AuNP and Iso Leu AuNP have similar hydrophobicity, the amount of lipoprotein on Leu AuNP was higher than Iso Leu AuNP (18.6% vs 14.1%), suggesting factors other than hydrophobicity contribute to their adsorption to the NPs. Lipoproteins are primarily divided into high-density lipoproteins (HDL), low-density lipoproteins (LDL), and very low-density lipoproteins (VLDL), whose components are apolipoproteins and lipids. LDL and VLDL are readily taken up by macrophages via different pathways, contributing to NP clearance mechanisms.^48^

Apolipoprotein B-100 (Apo B-100), a significant constituent of LDL proteins, was the most abundant protein in the corona of Leu and Iso Leu AuNPs, with ∼5 times more enrichment in the coronae than in human plasma. Leu AuNPs had 1.5 ± 0.8 (CI: 0–3.8) fold more Apo B-100 on their surface than Iso Leu AuNPs, highlighting the impact of surface chemistry on protein adsorption (Figure 5D and Table S4). Similarly, ApoE showed ∼11-fold enrichment on the Leu and Iso Leu AuNP compared to plasma with a higher preference for the Iso Leu AuNP surface. ApoE and ApoB are the primary apoproteins in VLDL, LDL, and chylomicrons. Bare AuNPs have been reported to show a preferred enrichment of apolipoprotein B-100, an observation in line with our findings.^49^ Our data extend these observations: replacing leucine with isoleucine amino acid reduces surface lipophilicity just enough to modulate Apo B loading. The more lipophilic Leu-AuNPs bind more Apo B (and other apolipoproteins) than their Iso-Leu counterparts, confirming that even subtle shifts in surface chemistry can reshape the composition of the corona and thereby influence downstream biological responses.

The percentage enrichment of complement proteins in the PC was similar on both NPs (Figures 5E and S3), with complement proteins C3 and C4–B being the most abundant. However, C4–B and C3 proteins were at least ∼1.1 ± 0.1­(CI: 0.84–1.40) folds higher on Leu AuNP than on Iso Leu AuNP. The complement C3 proteins enable macrophages to detect them via the appropriate complement receptors, such as complement receptors 1 and 3, resulting in early clearance of NP from the system.^50^ Notably, C3/C5 convertase was present in the PC of the Iso Leu AuNP but absent in the PC from Leu AuNPs. The C3 convertase is critical for classical and alternative complement activation pathways and cleaves C3 into its components that propagate the pathway. While both PCs contained C3 and C5, they may not induce the complement pathway to similar levels.

Immunoglobulins aid NP clearance by activating macrophage phagocytosis via Fc gamma receptors.^51^ Immunoglobulin’s abundance in the coronae was de-enriched on Iso Leu AuNP, while it was enriched 1.2-fold on Leu AuNP (Figures 5F and S3). Although Saha, reported a positive correlation between hydrophobicity and immunoglobulin adsorption on the NP surface,^34^ our results suggest that the protein corona is influenced by more than hydrophobicity alone. In this study, the difference in molecular arrangement, rather than overall hydrophobic character, modulated the individual enrichment of immunoglobulins.

### The PC Forms by Protein–Protein Interactions and Direct Interaction with the NP Surface

While an initial interaction between the NP surface and proteins in the surrounding media initiates the protein corona formation, it grows by protein–protein interactions, assembling a complex structure around the NP. The interactions between the different protein components and how the absence of specific proteins could alter the formation of the PC were analyzed using STRING proteomic analyses software. The proteins in the cluster were manually color-coded based on their biological function. The analysis showed that because the overall distribution of the proteins was similar, their clustering was generally similar (Figure 6A,B and Table S6). It was observed that for both Leu and Iso Leu AuNPs, the different clusters integrated into one mega-cluster through connections maintained by albumin (ALB), fibronectin (FN), and vitronectin (VTN). Interestingly, complement proteins segregated from the mega-cluster, forming an independent group. There were notable variations in the protein interactions caused by the absence of specific proteins, such as heparan sulfate glycan 2, apolipoprotein A2, and the presence of complement C6 proteins in Iso Leu AuNP PC. The presence or absence of these proteins resulted in a 20% difference in the number of interactions. The downstream consequence of the absence of such a difference is significant.

**Figure 6:**
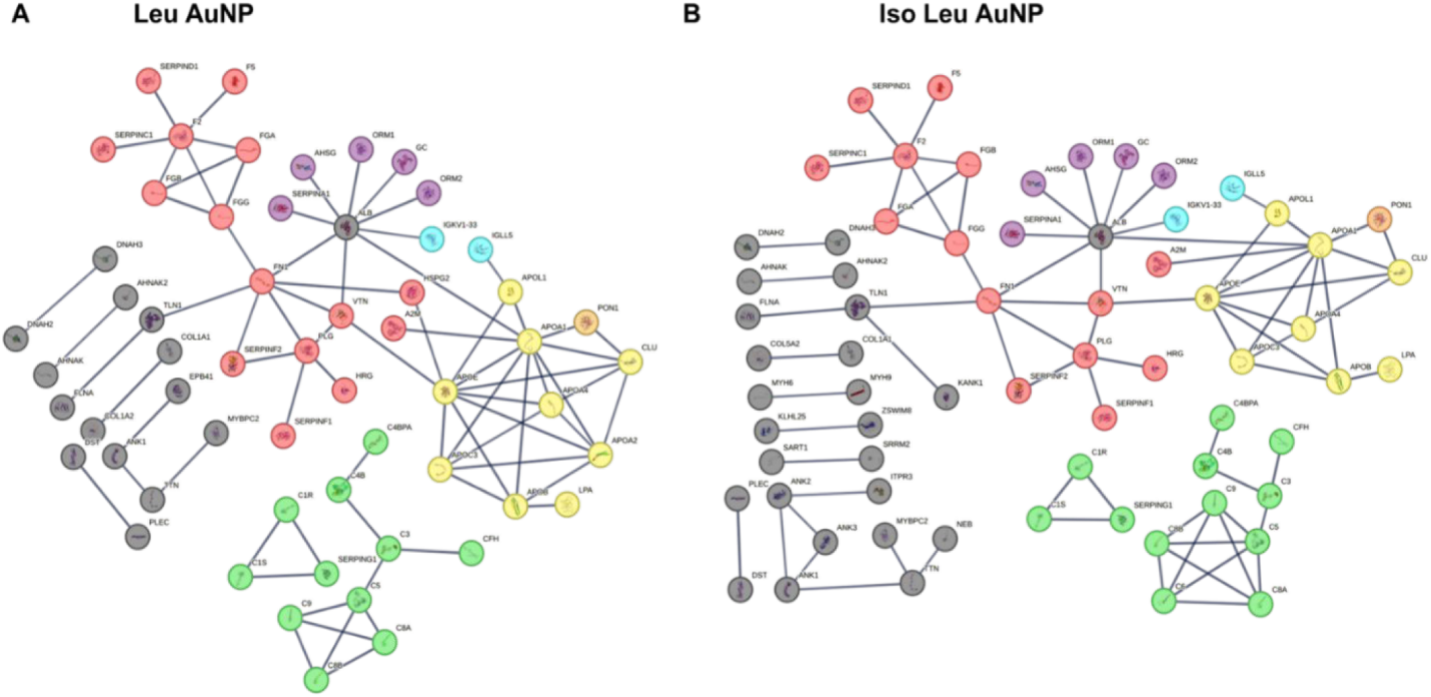
Predicted protein–protein interaction, generated by STRING software, of the proteins identified by LC-MS/MS analysis in (A) Leu AuNP PC and (B) Iso Leu AuNP PC. Protein–protein interactions were simulated between all the proteins identified in the corona, organized into clusters based on immune functions. Red: coagulation, purple: acute phase, yellow: lipoproteins, green: complement proteins, blue: immunoglobulins and black: nonimmune proteins. The list of genes in each cluster is provided in the Supporting Information.

The interaction between heparan sulfate glycan 2 and Apo E found in Leu AuNP PC leads to lipid accumulation in macrophages, increasing inflammatory cytokine production.^52^

### Isomeric Differences in Surface Chemistry Alter NP–PC Interactions with Macrophages

To understand the ramifications of surface-chemistry-induced changes in the protein corona on macrophages, we assessed their viability, uptake of the NP-protein corona complex, and cytokine release in their presence.

The toxicity was measured using a lactate dehydrogenase (LDH) assay, which measures the release of LDH into the culture media as the cell membrane loses integrity. The data are presented as the percent increase or decrease in toxicity of cells after exposure to NPs or NP-protein corona complexes relative to untreated dTHP-1 cells. While the pristine NPs did not affect cell viability, the NP-protein corona complexes were cytotoxic. However, there were no significant differences in the LDH percentage between the two protein corona-treated cells, suggesting that the difference in the protein corona did not produce an observable difference in cytotoxicity. This implies that the proteins in the corona could alter the macrophage viability, plausibly due to the presence of proteins such as immunoglobulins and complement proteins (Figure 7A). Extensive studies of the NP–PC interactions have shown that the PC can increase or decrease cytotoxicity. The composition of the protein corona, interaction between the proteins in the corona, and conformation of the proteins are significant factors affecting the toxicity of NPs to cells. While the protein corona reduces cytotoxicity toward lung tumor cells and HeLa cells, the cytotoxicity of some NPs to mouse macrophages increases in the presence of the PC.^53^ In this study, the protein corona increased the toxicity of amino acid-conjugated AuNPs to dTHP-1 macrophages. The implications of the protein corona differences on macrophage interaction were further explored by assessing the endocytosis of the AuNP PCs by macrophages. The cellular uptake of the NP-protein corona complex was visualized using fluorescence microscopy (Figure 7B,C) and quantified using inductively coupled plasma-mass spectrometry (ICP-MS) (Figure 7D). The fluorescence microscopy showed the association of the NP-PC complex with the cells, wherein the NP-protein corona presented as aggregate mass on the cell surface rather than as individually dispersed NPs. A negative control with no NP treatment showed no red intensity and was used as a baseline for further MFI calculation (Figure S4). The mean fluorescence intensity (MFI) of the nanoparticle–protein corona (NP-PC) complex associated with macrophages was significantly higher for the Leu AuNPs than for their Iso leucine counterparts (Figure 7C). ICP-MS confirmed a similar trend: cells exposed to Leu AuNP PCs contained 10.5 ± 4.5 pg of Au per cell, whereas those treated with Iso-Leu-AuNP PCs contained 6.1 ± 2.8 pg (*n* = 3). Although this ∼1.7-fold difference did not reach statistical significance (*t*-test, *p* = 0.21), the direction and magnitude mirrored the fluorescence data and are likely masked by the small sample size and biological variability. Taken together with the parallel cytotoxicity results, these findings indicate that Leu AuNP PCs could be more readily internalized than Iso Leu AuNP PCs, underscoring how subtle differences in surface chemistry can reshape the protein corona and, in turn, modulate macrophage uptake.

**Figure 7:**
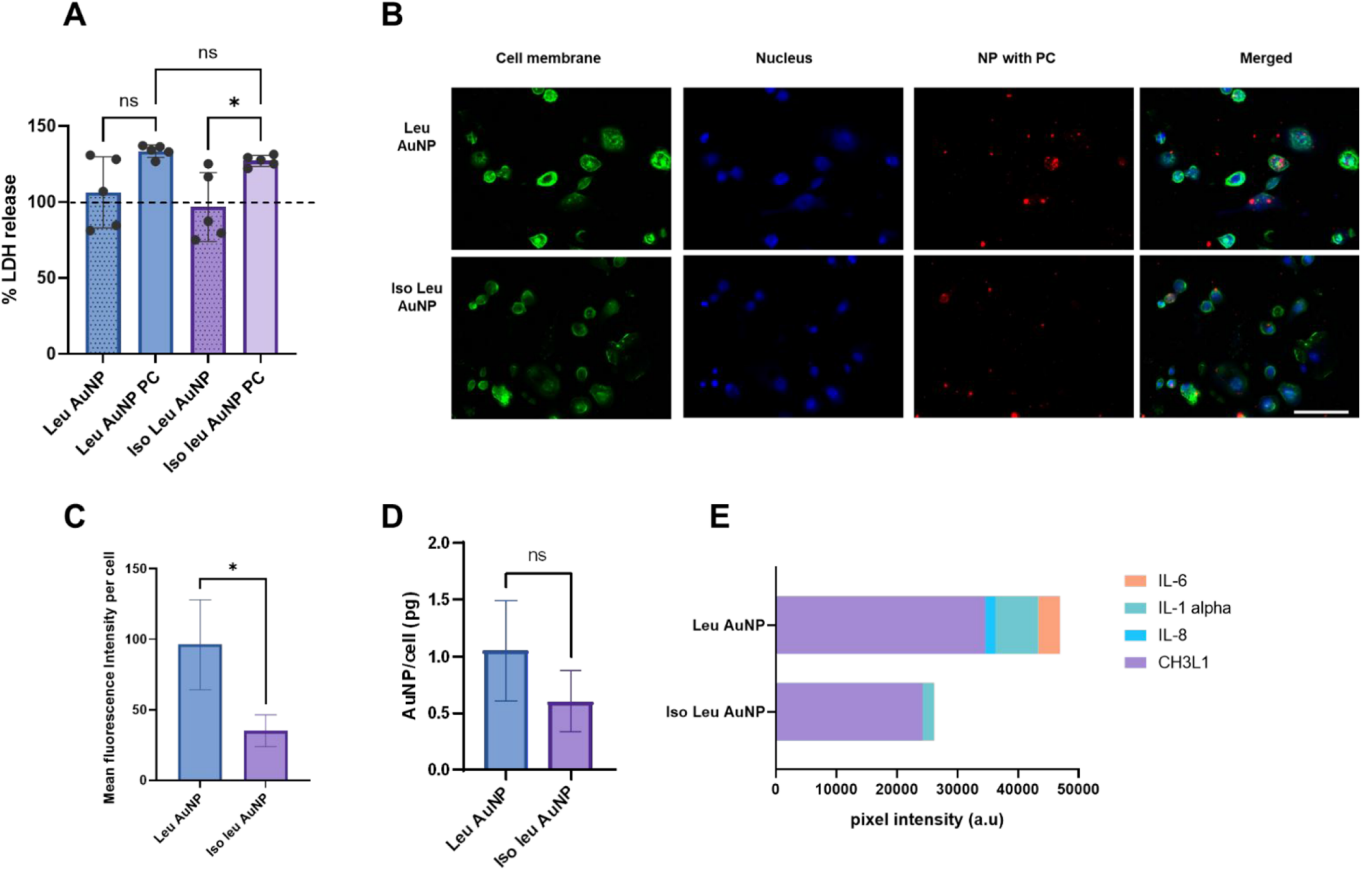
NP-PC interaction with THP-1 macrophage cells. (A) LDH assay to measure the cytotoxicity of pristine nanoparticles and nanoparticle–protein corona complexes. The line indicates LDH released from cells only, taken as 100% (one-way ANOVA followed by post hoc Tukey test, *n* = 3, *p < 0.05). (B) Fluorescence image of AuNP PC uptake. Green = cell membrane, blue= cell nucleus, red = AuNP PC. (C) Mean fluorescent intensity of AuNP PC associated with macrophages (Student’s *t*-test, *n* = 3, *p < 0.05). (D) Nanoparticle uptake by macrophage cells measured using ICP-MS (Student’s *t*-test, *n* = 3, *p < 0.05). (E) Inflammatory cytokines released from macrophages in response to AuNP PC treatment (Scale bar= 100 μm).

The NP-PC complex’s internalization can affect inflammatory cytokine production through MAPK or NF-κB pathways.^54^ The downstream effect of the activation of the NF-κB pathway is the release of pro-inflammatory cytokines.^55^ Interaction of NPs with cells can trigger the production of inflammatory cytokines such as IL-6, IL-1 alpha, CH3L1, and IL-8.^56,57^ The secretion of these cytokines by macrophages after exposure to AuNP-PC was measured by ELISA (Figure 7E and Table S6). Macrophages exposed to Leu AuNP-PC secreted 3.6-fold more IL-1 alpha than Iso Leu AuNP-PC. The macrophages produced IL-6 upon interaction with Leu AuNP PC, whereas both treatments secreted IL-1 alpha. The increased production of these inflammatory cytokines by macrophages after exposure to Leu AuNP-PC agrees with the increased complement pathway association presented by the Reactome Pathway Analysis (Table 1). IL-1 alpha inflammatory cytokines are produced when the toll-like receptors (TLRs) are activated by molecules such as lipoproteins, peptidoglycans, and fibronectins.^58^ The pathways activated by the PC (Table 1) show equal enrichment of pathways associated with the activation and regulation of TLRs by endogenous ligands. CH3L1 is an inflammatory cytokine produced by macrophages and has been correlated with macrophage proliferation and angiogenesis.^59,60^ The downstream effect of CH3L1 activation is the production of IL-8, a cytokine associated with T-cell and neutrophil activation, resulting in systemic inflammation and rapid elimination of the NPs from circulation.^59^ We observe that while Leu PC- and Iso Leu PC-treated macrophages produce CH3L1 cytokines, the amount produced from Leu AuNP-PC-treated macrophages is 1.5 times higher than that from Iso Leu AuNP-PC, with a notable production of IL-8 cytokines in Leu AuNP-PC. IL-8 production was absent from macrophages with the Iso Leu AuNP-PC treatment. The exclusive production of IL-8 from Leu AuNP-PC emphasizes the influence of nuanced differences NPs’ surface chemistry on immunomodulation and its potential impact on NPs’ fate *in vivo*.

**Table 1:** List of Significantly Enriched Pathways Contributing to NP Clearance Identified in Each Protein Corona.^a^

Pathways	Leu AuNP	Iso Leu AuNP
Regulation of Complement cascade	19.29	19.43
Complement cascade	18.63	18.74
Initial triggering of complement	8.21	7.67
Terminal pathway of complement	7.36	8.75
Creation of C4 and C2 activators	6.30	4.85
Scavenging by class A receptors	5.87	4.24
CD22-mediated BCR regulation	5.59	4.20
FCGR3A-mediated IL10 synthesis	5.50	4.17
FCGR activation	5.32	3.97
FCGR3A-mediated phagocytosis	4.95	3.70
Role of phospholipids in phagocytosis	4.86	4.50
Antigen activates B cell receptor (BCR)	4.77	4.41
Fcgamma receptor (FCGR) dependent phagocytosis	4.35	3.96
Regulation of TLR by endogenous ligand	4.31	4.09
Activation of C3 and C5	4.20	5.63
MyD88 deficiency (TLR2/4)	3.25	3.08
Signaling by the B Cell Receptor (BCR)	3.19	2.86
IRAK4 deficiency (TLR2/4)	3.18	3.02
Scavenging by class B receptors	2.86	2.75

a Values depict the negative log 10 of the *p*-value.

Since Leu and Iso Leu AuNPs showed differences in the macrophage interaction due to protein corona composition, we performed pathway analysis using the Reactome Pathway database to understand the biological implications of the corona proteins.^61^ While the analysis does not consider the abundance of individual proteins, the database evaluates the over-representation of proteins associated with specific pathways in a data set. The pathway analysis showed the over-representation of proteins associated with complements, immunoglobulins, and lipoproteins (Table 1).

Pathways regulating and activating the complement cascade were equally enriched on Leu and Iso Leu AuNPs. The complement pathway involves foreign body recognition and promotes cell death by forming membrane channels.^62^ The generation of C4 and C2 activators, a step unique to the classical complement pathway, is 22% higher in Leu AuNP than iso Leu AuNP. However, Iso Leu AuNP shows higher enrichment of the pathway activation of C3 and C5, a hallmark of the alternative pathway.^63^ Therefore, it could be suggested that the Leu AuNP has a greater tendency to induce the classical complement pathway, while the Iso Leu AuNP favors the alternative pathway. The pathway data correlate with the observed toxicity data in macrophages.

Furthermore, the pathway data also explain the differences in the uptake profile between the protein corona-coated NPs. Leu AuNP shows ∼22% more IgG and IgM proteins than iso Leu AuNP adsorbed onto its surface. These proteins are responsible for activating phagocytosis pathways mediated through Fc gamma receptors and scavenging by class A receptors.^64–66^ Subsequently, both these pathways are ∼1.3-fold higher in the proteins adsorbed on Leu AuNP than Iso Leu AuNP, correlating with the 1.7-fold higher macrophage uptake observed in Leu AuNP. These results agree with the behavior observed in macrophages when exposed to protein corona-coated amino acid-conjugated NPs, triggering uptake through phagocytosis.^64^

Leucine and isoleucine—together with valine—are the most common hydrophobic residues in transmembrane domains, where they help maintain membrane integrity.^67^ Their strong lipid affinity also makes them attractive surface modifiers for gene- and drug-delivery nanoparticles. Notably, leucine is slightly more lipophilic and more disruptive to ordered lipid domains than isoleucine.^68^ When we conjugate these residues onto gold nanoparticles, a subtle difference is preserved: Leu-AuNPs recruit slightly more apolipoprotein B-100 and other lipoproteins than Iso-Leu-AuNPs. This compositional shift has functional consequences. Lipoprotein-rich coronas are recognized by macrophage scavenger receptors and can drive foam-cell formation—an outcome seen here by the higher pro-inflammatory cytokine release we observe for Leu-AuNP-PC treatment. The results underscore a key design rule: even a methyl shift can reshape the protein corona and reprogram the immune response. Thorough in vitro corona profiling should therefore be a mandatory step when tailoring nanoparticle surfaces for specific biomedical applications.

## Conclusion

The study presented here explores the protein corona’s susceptibility to subtle changes in NPs’ surface chemistry. Using Leu- and Iso-leu-modified AuNPs, we demonstrate that the protein corona is influenced by isomeric changes in the surface chemistry of the NPs, with implications for macrophage interaction and immune responses in vitro. We demonstrate that isomeric differences in surface chemistry yielded a 31% change in the lipoproteins, 16% change in immunoglobulins, and 4% change in complement, coagulation, and acute phase proteins. These results suggest that charge alone is not sufficient to explain the differences in the protein corona observed on NPs; the difference in surface chemistry also contributes. These differences led to a 1.7-fold difference in macrophage uptake, with higher uptake directly correlating to higher inflammatory cytokine production, potentially affecting NPs’ fate in vivo. This is one of the few studies that have successfully delineated the role of surface chemistry on the PC without the impact of the surface charge or hydrophobicity. The particle system developed here provides a strategy to investigate the contributions of surface chemistry to the evolution of the protein corona.

## Materials and Methods

### Materials

Tetra-chloroauric­(III) acid trihydrate, acetyl-l-leucine, and acetyl-l-isoleucine were purchased from Thermo Scientific Chemicals (Waltham, MA). Poly­(allylamine) hydrochloride was purchased from Sigma-Aldrich (St. Louis, Missouri). NH2-PEH-SH, MW200, was purchased from Laysan Bio Inc. (Arab, AL). *N*-(3-(Dimethylamino)­propyl)-*N*’-ethylcarbodiimide hydrochloride was purchased from VWR, and *N*-hydroxysuccinimide was purchased from Alfa Aesar (Haverhill, MA). Human plasma with the K2 EDTA anticoagulant was purchased from Innovative Research (Novi, MI). Polyurethane tubing and fittings of the desired size and shape were purchased from the United States Plastic Corps (Lima, OH).

### Synthesis of Amino Acid Conjugated Gold Nanoparticles

Amine-modified gold NPs were synthesized by reducing tetra-chloroauric (III) acid trihydrate with poly­(allylamine) hydrochloride (PAH). In a typical reaction, a 100 mL solution of 10 mM tetra-chloroauric (III) trihydrate in deionized water was heated to 100 °C, to which 200 μL of 1 mg/mL PAH was added. The mixture was allowed to interact for 30 min until the solution turned pink, which was then removed from heat and allowed to cool to room temperature. Once cooled, 1 mg of NH_2_–PEG-SH (2000 Da MW) was added to the solution and stirred for 1 h. The amino acids were conjugated to gold NPs via EDC-NHS conjugation chemistry. The amino acids were activated by adding 25 mM of *N*-(3-(dimethylamino)­propyl)-*N*’-ethylcarbodiimide hydrochloride (EDC), VWR) and *N*-hydroxysuccinimide (NHS) to 5 mL of 10 mM amino acids for 30 min. The mixture was then mixed with the PEGylated gold NP solution and allowed to interact for 1 h. The synthesized amino acid-modified gold NPs were recovered by centrifugation for 45 min at 2831g. (Eppendorf 5804R Series)

### Dye Conjugation to Nanoparticle Surface

The NP-dye conjugation was performed to quantify the extent of surface coverage by the amino acids. Briefly, 1 × 10^11^/ml of the NPs were incubated with 1 μg of the Alexa Fluor 594 NHS ester (ThermoFisher Scientific, Waltham, MA) dye for 2 h. The dye-conjugated NPs were recovered by centrifugation. The amount of dye conjugated to the NPs was measured using fluorescence spectroscopy. A standard curve of known dye concentrations and corresponding fluorescence intensities was plotted to determine the amount of dye conjugated to each NP surface.

### Protein Corona Formation on Nanoparticles

Human plasma from a healthy donor was procured from Innovative Research, which has been approved for in vitro laboratory applications. The demographic profile of the plasma donor indicated Hispanic female origin.

Human plasma was centrifuged at 1000 rpm to remove protein aggregates before use. Flow conditions for plasma proteins were established using a peristaltic pump (Fisherbrand Variable-Flow Peristaltic Pumps) connected to an 8-in. length polyurethane tube with a diameter of 3/32 in. The system was filled with 100% human plasma (2 mL) and set to a 25 mL/min flow rate. The NPs (1 × 10^11^/mL 50 μL) were injected in the plasma flow direction using an insulin syringe and allowed to circulate for 10 min at 37 °C. After 10 min, the NPs were recovered from the plasma by centrifugation (Thermo Scientific Sorvall Legend Micro 21 Microcentrifuge) at 21,000*g* for 15 min. The recovered NPs were washed in phosphate-buffered saline (PBS) three times to remove the unbound and soft corona with centrifugation recovery after each wash.

### Characterization of Nanoparticles

Hydrodynamic diameter, polydispersity index , and zeta potential measurements were performed on a Malvern Zetasizer Nano instrument on particles resuspended in ultrapure water at room temperature. Transmission electron microscopy (TEM) was performed to confirm the size distribution and morphology of plasma-treated and untreated NPs using a JEOL 1200EX. NPs were drop-cast onto copper grids and air-dried at room temperature before imaging. NP-protein corona complexes were stained with uranyl acetate to enhance the protein’s contrast. The amount of protein associated with the NP pellets after washing was measured by micro-BCA assay (ThermoFisher, Waltham, MA) following the manufacturer’s instructions.

#### Fourier Transform Infrared (FTIR) Spectroscopy:

FTIR spectra were recorded on a Bruker Alpha equipped with a diamond-ATR crystal to verify amide bond formation on the nanoparticle surface after amino acid conjugation. Briefly, 2 μL of a nanoparticle suspension was drop-cast onto the ATR crystal, and spectra were collected from 4000 to 400 cm^– 1^ using OPUS software. All spectra were baseline-corrected and normalized prior to comparison.

#### UV–Visible Spectroscopy:

The optical properties of the nanoparticles were measured with a Tecan Infinite 200 PRO M Plex plate reader operated in the absorbance mode. A 50 μL aliquot of the nanoparticle suspension was diluted 1:12 (v/v) in deionized water and dispensed into a UV-transparent, flat-bottom 96-well plate. Absorbance spectrum was collected from 400 to 800 nm, and each spectrum was baseline-corrected and normalized to allow direct comparison between samples.

### Liquid Chromatography–Mass Spectrometry (LC-MS/MS)

Srotein digestion was carried out by S-Trap sample processing technology (S-TRAP) according to the protocol. Briefly, a reductant (1 μL) was added to protein (5 μg) dissolved in 1X lysis buffer. After incubation for 15 min at 55 °C, an alkylator (1 μL) was added, and the solution was incubated for 10 min. The pH of the solution was adjusted to 1 with acid and digested overnight at 37 °C. The peptides were eluted using elution buffer, dried, and resuspended to the desired LC-MS/MS analysis volume.

#### LC-MS/MS:

LC-MS/MS analysis was performed using a Thermo Scientific UltiMate 3000 nanoUHPLC system coupled to an Orbitrap Fusion mass spectrometer. Dried samples were reconstituted in a mixture containing 98% water, 2% acetonitrile, and 0.1% formic acid (20 μL), and the injection volume was set to 1 μL. Samples were separated with an Acclaim PepMap C18 column (0.075 mm × 150 mm, 2 Å particle size) at a 0.400 μL/min flow rate. The mobile phase consisted of water, acetonitrile, and formic acid (98/2/0.1 for buffer A and 2/98/0.1 for buffer B). The gradient was as follows: equilibration at 2% B for 5 min, ramping up to 45% B at 37 min, 90% B from 40 to 46 min, and re-equilibration at 2% B from 47 to 60 min.

Eluted peptides were introduced into the mass spectrometer by positive ESI at 2450 V, and the ion transfer tube temperature was set to 275 °C. Data were obtained in top-speed mode with a cycle duration of 3 s. Full scans were acquired in the Orbitrap at a resolution of 120,000 at *m*/*z* 200. The mass range, RF lens amplitude, and maximum injection time were 400–1600 *m*/*z*, 60%, and 100 ms, respectively. MS/MS spectra were acquired in the ion trap set to a rapid scan rate. The precursor isolation window was set to 1.6 *m*/*z*. Fragmentation was achieved by HCD at a fixed collision energy of 28%. Dynamic exclusion was set to 60 s with a tolerance of 10 ppm. Data were processed using Thermo Scientific Proteome Discoverer v. 2.4. A custom theoretical fragment database was derived from the human reference proteome available from UniProt (UP000005640, 78120 protein sequences). Spectra were processed and matched against the database using the basic SequestHT processing and consensus workflows included with the software and tailored for the tribrid platform, with the precursor and fragment tolerances set to 5 ppm and 0.6 Da, respectively. Two peptides were required for the successful identification of a protein. The abundance percentage of each protein was calculated by dividing each peptide number by the sum of the peptides in that particular group. The abundance percentage thus calculated was used for all of the analyses.

### Protein–Protein Interaction

The accession numbers of the proteins identified from LC-MS/MS were queried into STRING software as a group to map the protein–protein interactions in the protein corona. Constraining parameters allowed the display of only those physical interactions that had a high confidence (0.7) interaction score. MCL clustering was used to cluster the proteins,^69^ generating clusters in each corona.

#### Pathway analysis:

Reactome pathway database software was utilized to generate pathways enriched in the protein corona. The *p*-values generated by the software compared the pathway enrichment among the experimental groups.

### THP-1 Cell Culture

THP-1 monocyte cell lines were grown and maintained in RPMI supplemented with 10% fetal bovine serum (v/v), 1% penicillin-streptomycin, and 55 mM beta-mercaptoethanol in a 5% CO2 incubator at 37 °C. To differentiate the suspended THP-1 cells to adherent M0 macrophages, the cells were seeded in a 96-well plate at a seeding density of 20,000 cells in 5 μg/mL of Phorbol 12-myristate 13-acetate (Adipogen, CA).^70^ The media were changed on day 3, and the cells were ready to be used on the fifth day postseeding.

### Lactate Dehydrogenase Assay

M0 macrophages generated from THP-1 cells were seeded at a density of 20,000 cells/ml in a 96-well plate and allowed to attach overnight. Cell toxicity was assessed 24 h after incubation with 1 × 10^10^/ml of NPs with and without PC in serum-free media using the Lactate Dehydrogenase assay (CytoTox 96 Non-Radioactive Cytotoxicity Assay) following the manufacturer’s protocols. Briefly, 50 μL of the media from the cells was removed and incubated with an equal volume of CytoTox96 reagent assay. The assay reagent and media were incubated for 30 min at room temperature. 50 μL of the stop solution was added, and the absorbance was measured at 490 nm using a Biotek Cytation 5 (Agilent Technologies, USA) plate reader.

### Cellular Uptake of NP–PC Complex

Undifferentiated macrophages were plated in a 6-well plate at a seeding density of 1 million cells and differentiated according to the method given earlier. The macrophages were treated with 1 × 10^10^ NPs, specifically AuNPs with protein corona and allowed to interact for 6 h, after which the supernatant containing the NPs was removed. The cells were washed with 1X PBS to remove unbound NP- protein aggregates. The cells were then treated with trypsin to detach them from the well plate and resuspended in RPMI media supplemented with fetal bovine serum (FBS) to inactivate the trypsin. The cells were washed three times using 1X PBS, resuspended in 1x phosphate-buffered saline (PBS), and counted using a hemocytometer. Finally, the cells were submitted to the ICP-MS facility for further analysis.

#### Fluorescent Imaging:

After incubation in plasma under the flow conditions and washing in PBS to remove the unbound proteins, the PC-coated AuNPs were fluorescently labeled with Alexa Fluor 594 NHS ester (ThermoFisher Scientific, Waltham, MA). The NPs were sonicated for 5 min in a bath sonicator (VWR Ultrasonic Cleaners) to break down aggregates before being added to cells. The protein corona-coated NPs (1 × 10^10^/mL) were incubated with dTHP-1 cells at a seeding density of 10 ,000 cells per well in a 96-well plate, in serum-free media for 6 h. The cells were washed three times with phosphate-buffered saline (PBS), stained with Hoechst 33258 and Alexa Fluor 488 Phalloidin (ThermoFisher Scientific, Waltham, MA) dye, and fixed in 4% paraformaldehyde before imaging. The washed cells were imaged using a Lionheart LX automated microscope (Biotek, Winooski, VT) using the filter cubes corresponding to the Texas Red channel to image NPs, DAPI channel to image cell nuclei, and GFP channel to image the cell membrane. Images were taken in the fluorescence setting with focus on the cell membrane to visualize the NP-PC complex. Mean fluorescence intensity (MFI) was quantified in ImageJ by measuring the red signal associated exclusively with cellular regions; background fluorescence was subtracted to obtain the true MFI.

### Cytokine Assay

Cytokine production from cells after treatment with NP-protein corona complex was assessed via Proteome Profiler Human XL Cytokine Array Kit (R&D systems, MN) according to manufacturer’s protocol. Of the 105 cytokines measured by the kit, only the relevant cytokines have been reported in this study.

### Statistics

GraphPad PRISM version 9.1 (La Jolla, CA) was used to perform statistical analyses, with the error bars denoting standard deviation. Statistical comparisons were determined by either one-way ANOVA followed by Tukey’s multiple comparison test or Student’s *t*-test as required, and the outcomes are denoted as follows: *p < 0.05; **p < 0.01; ***p < 0.0001; ns: not significant. The number of trials is three unless specifically mentioned otherwise.

## Supplementary Material





## Data Availability

All the data supporting the findings in this manuscript are provided in the supplementary file. The mass spectrometry proteomics data have been deposited to the ProteomeXchange via PRIDE^71^ partner repository with the data set identifier PXD051722 and 10.6019/PXD051722.

## Notes

Supplementary Tables: list of all proteins (Table S1), top 10 proteins (Table S2), functional groups in the heatmap (Table S3 and S4), STRING analysis and clustering (Table S5), and cytokine heat map (Table S6) (XLSX)
Supplementary Figures: Intensity, number, and volume distribution of Leu AuNP PC and Iso Leu AuNP PC (Figure S1), comparison of number and intensity distribution before and after protein corona formation on Leu AuNP and Iso Leu AuNP (Figure S2), zeta potential kinetics of NP-PC over time (Figure S3), and dTHP macrophages only without NP treatment (Figure S4) (PDF)

## References

[1] Veider F., Armengol E. S., Bernkop-Schnürch A. (2023). Charge-Reversible
Nanoparticles:
Advanced Delivery Systems for Therapy and Diagnosis. Small.

[2] de
Lazaro I., Mooney D. J. (2021). Obstacles and opportunities in a
forward vision for cancer nanomedicine. Nat.
Mater..

[3] Sadauskas E., Wallin H., Stoltenberg M., Vogel U., Doering P., Larsen A., Danscher G. (2007). Kupffer cells are central in the
removal of nanoparticles from the organism. Part. Fibre Toxicol..

[4] Tang H. (2023). Cholesterol modulates
the physiological response to nanoparticles
by changing the composition of protein corona. Nat. Nanotechnol..

[5] Yu Q., Zhao L., Guo C., Yan B., Su G. (2020). Regulating
Protein Corona Formation and Dynamic Protein Exchange by Controlling
Nanoparticle Hydrophobicity. Front. Bioeng.
Biotechnol..

[6] Saptarshi S. R., Duschl A., Lopata A. L. (2013). Interaction
of nanoparticles with
proteins: relation to bio-reactivity of the nanoparticle. J. Nanobiotechnol..

[7] Gonzalez-Garcia L. E. (2022). Nanoparticles Surface Chemistry Influence on Protein Corona Composition
and Inflammatory Responses. Nanomaterials.

[8] Kurtz-Chalot A. (2017). Impact of silica nanoparticle
surface chemistry on protein corona
formation and consequential interactions with biological cells. Mater. Sci. Eng., C.

[9] Hristov D. R., Rocks L., Kelly P. M., Thomas S. S., Pitek A. S., Verderio P., Mahon E., Dawson K. A. (2015). Tuning
of nanoparticle
biological functionality through controlled surface chemistry and
characterisation at the bioconjugated nanoparticle surface. Sci. Rep..

[10] Qie Y. (2016). Surface modification
of nanoparticles enables selective evasion of
phagocytic clearance by distinct macrophage phenotypes. Sci. Rep..

[11] Ravishankar S., Nedumaran A. M., Gautam A., Ng K. W., Czarny B., Lim S. (2023). Protein nanoparticle
cellular fate and responses in murine macrophages. NPG Asia Mater..

[12] Gattu R., Ramesh S. S., Nadigar S., Gowda D. C., Ramesh S. (2023). Conjugation
as a Tool in Therapeutics: Role of Amino Acids/Peptides-Bioactive
(Including Heterocycles) Hybrid Molecules in Treating Infectious Diseases. Antibiotics.

[13] Dodds J. N., May J. C., McLean J. A. (2017). Investigation
of the Complete Suite
of the Leucine and Isoleucine Isomers: Toward Prediction of Ion Mobility
Separation Capabilities. Anal. Chem..

[14] Biro J. C. (2006). Amino acid
size, charge, hydropathy indices and matrices for protein structure
analysis. Theor. Biol. Med. Model.

[15] Pliska V., Schmidt M., Fauchère J.-L. (1981). Partition
coefficients of amino acids
and hydrophobic parameters π of their side-chains as measured
by thin-layer chromatography. J. Chromatogr..

[16] Ojea-Jimenez I., Garcia-Fernandez L., Lorenzo J., Puntes V. F. (2012). Facile preparation
of cationic gold nanoparticle-bioconjugates for cell penetration and
nuclear targeting. ACS Nano.

[17] Goodman C. M., McCusker C. D., Yilmaz T., Rotello V. M. (2004). Toxicity of gold
nanoparticles functionalized with cationic and anionic side chains. Bioconjugate Chem..

[18] Chen C., Constantinou A., Deonarain M. (2011). Modulating antibody pharmacokinetics
using hydrophilic polymers. Expert Opin. Drug
Del..

[19] Ji Y. (2020). DFT-Calculated
IR Spectrum Amide I, II, and III Band Contributions
of N-Methylacetamide Fine Components. ACS Omega.

[20] Cho I. K., Chang C. L., Li Q. X. (2013). Diet-induced
over-expression of flightless-I
protein and its relation to flightlessness in Mediterranean fruit
fly, Ceratitis capitata. PLoS One.

[21] Talat M., Singh A. K., Srivastava O. N. (2011). Optimization
of process variables
by central composite design for the immobilization of urease enzyme
on functionalized gold nanoparticles for various applications. Bioprocess Biosyst. Eng..

[22] Dai Q., Walkey C., Chan W. C. W. (2014). Polyethylene
Glycol Backfilling Mitigates
the Negative Impact of the Protein Corona on Nanoparticle Cell Targeting. Angew. Chem., Int. Ed..

[23] Li X., Jiang L., Zhan Q., Qian J., He S. (2009). Localized
surface plasmon resonance (LSPR) of polyelectrolyte-functionalized
gold-nanoparticles for bio-sensing. Colloids
Surf., A.

[24] Koushki E., Mowlavi A. A., Hoseini S. T. (2023). Application of Localized Surface
Plasmon Resonance of Conjugated Gold Nanoparticles in Spectral Diagnosis
of SARS-CoV-2: A Numerical Study. Plasmonics.

[25] Ma Y., Hong J., Ding Y. (2020). Biological
Behavior Regulation of
Gold Nanoparticles via the Protein Corona. Adv.
Healthcare Mater..

[26] Han Q. (2025). Silica Nanoparticle-Protein Aggregation and Protein Corona Formation
Investigated with Scattering Techniques. ACS
Appl. Mater. Interfaces.

[27] Piella J., Bastus N. G., Puntes V. (2017). Size-Dependent Protein-Nanoparticle
Interactions in Citrate-Stabilized Gold Nanoparticles: The Emergence
of the Protein Corona. Bioconjugate Chem..

[28] Dridi N., Jin Z., Perng W., Mattoussi H. (2024). Probing Protein Corona Formation
around Gold Nanoparticles: Effects of Surface Coating. ACS Nano.

[29] Chen K. (2011). Electrostatic selectivity in protein-nanoparticle interactions. Biomacromolecules.

[30] Leitner N. S., Schroffenegger M., Reimhult E. (2021). Polymer Brush-Grafted Nanoparticles
Preferentially Interact with Opsonins and Albumin. ACS Appl. Bio Mater..

[31] Papini E., Tavano R., Mancin F. (2020). Opsonins and Dysopsonins of Nanoparticles:
Facts, Concepts, and Methodological Guidelines. Front. Immunol..

[32] Dobrovolskaia M. A. (2014). Protein corona composition does not accurately predict hematocompatibility
of colloidal gold nanoparticles. Nanomedicine.

[33] Ritz S. (2015). Protein corona of nanoparticles:
distinct proteins regulate the cellular
uptake. Biomacromolecules.

[34] Saha K. (2016). Regulation of Macrophage
Recognition through the Interplay of Nanoparticle
Surface Functionality and Protein Corona. ACS
Nano.

[35] Tenzer S. (2011). Nanoparticle Size Is a Critical Physicochemical Determinant of the
Human Blood Plasma Corona: A Comprehensive Quantitative Proteomic
Analysis. ACS Nano.

[36] Burnand D. (2018). Beyond Global Charge:
Role of Amine Bulkiness and Protein Fingerprint
on Nanoparticle-Cell Interaction. Small.

[37] Maity A., De S. K., Chakraborty A. (2021). Interaction
of Aromatic Amino Acid-Functionalized
Gold Nanoparticles with Lipid Bilayers: Insight into the Emergence
of Novel Lipid Corona Formation. J. Phys. Chem.
B.

[38] Caracciolo G. (2019). Disease-specific protein corona sensor arrays may have disease detection
capacity. Nanoscale Horiz..

[39] Sander L. E. (2010). Hepatic acute-phase
proteins control innate immune responses during
infection by promoting myeloid-derived suppressor cell function. J. Exp. Med..

[40] Papa E., Doucet J. P., Sangion A., Doucet-Panaye A. (2016). Investigation
of the influence of protein corona composition on gold nanoparticle
bioactivity using machine learning approaches. SAR QSAR Environ. Res..

[41] Janciauskiene S., Welte T. (2016). Well-Known and Less Well-Known Functions
of Alpha-1 Antitrypsin.
Its Role in Chronic Obstructive Pulmonary Disease and Other Disease
Developments. Ann. Am. Thorac. Soc..

[42] Chen G. J. (2011). Fibrinogen Clot Induced
by Gold-Nanoparticle In Vitro. J. Nanosci. Nanotechnol..

[43] Dobrovolskaia M. A. (2009). Interaction of colloidal
gold nanoparticles with human blood: effects
on particle size and analysis of plasma protein binding profiles. Nanomed-Nanotechnol..

[44] Farrell D. H. (2004). Pathophysiologic
roles of the fibrinogen gamma chain. Curr. Opin.
Hematol..

[45] Kharazian B. (2018). Bare surface of gold nanoparticle induces inflammation through unfolding
of plasma fibrinogen. Sci. Rep..

[46] Sasidharan A., Riviere J. E., Monteiro-Riviere N. A. (2015). Gold and
silver nanoparticle interactions
with human proteins: impact and implications in biocorona formation. J. Mater. Chem. B.

[47] Lima T., Bernfur K., Vilanova M., Cedervall T. (2020). Understanding
the Lipid and Protein Corona Formation on Different Sized Polymeric
Nanoparticles. Sci. Rep..

[48] Remmerie A., Scott C. L. (2018). Macrophages and lipid metabolism. Cell. Immunol..

[49] Jang G. J., Jeong J. Y., Kang J., Cho W., Han S. Y. (2021). Size Dependence
Unveiling the Adsorption Interaction of High-Density Lipoprotein Particles
with PEGylated Gold Nanoparticles in Biomolecular Corona Formation. Langmuir.

[50] Moghimi S. M., Simberg D. (2017). Complement activation turnover on
surfaces of nanoparticles. Nano Today.

[51] Daeron M. (2014). Fc Receptors
as Adaptive Immunoreceptors. Curr. Top Microbiol..

[52] Wilsie L. C., Chanchani S., Navaratna D., Orlando R. A. (2005). Cell surface heparan
sulfate proteoglycans contribute to intracellular lipid accumulation
in adipocytes. Lipids Health Dis..

[53] Wu G., Jiang C., Zhang T. (2018). FcgammaRIIB receptor-mediated apoptosis
in macrophages through interplay of cadmium sulfide nanomaterials
and protein corona. Ecotoxicol. Environ. Saf..

[54] Niu Y., Tang M. (2022). In vitro review of
nanoparticles attacking macrophages: Interaction
and cell death. Life Sci..

[55] Liu T., Zhang L., Joo D., Sun S. C. (2017). NF-kappaB signaling
in inflammation. Signal Transduction Targeted
Ther..

[56] Niu Y. R., Tang M. (2022). In vitro review of
nanoparticles attacking macrophages: Interaction
and cell death. Life Sci..

[57] Wang F. J. (2013). The biomolecular corona is retained during nanoparticle uptake and
protects the cells from the damage induced by cationic nanoparticles
until degraded in the lysosomes. Nanomed-Nanotechnol..

[58] Billack B. (2006). Macrophage
activation: role of toll-like receptors, nitric oxide, and nuclear
factor kappa B. Am. J. Pharm. Educ..

[59] Zhao T., Su Z., Li Y., Zhang X., You Q. (2020). Chitinase-3 like-protein-1
function and its role in diseases. Signal Transduction
Targeted Ther..

[60] Kawada M. (2012). Chitinase 3-like 1 promotes
macrophage recruitment and angiogenesis
in colorectal cancer. Oncogene.

[61] Griss J. (2020). ReactomeGSA - Efficient
Multi-Omics Comparative Pathway Analysis. Mol.
Cell. Proteomics.

[62] Corbo C. (2016). The impact of nanoparticle protein corona on cytotoxicity,
immunotoxicity
and target drug delivery. Nanomedicine.

[63] Kinoshita T. (1988). C5 convertase of the
alternative complement pathway: covalent linkage
between two C3b molecules within the trimolecular complex enzyme. J. Immunol..

[64] Yan Y. (2013). Differential roles of the protein corona in the cellular
uptake of
nanoporous polymer particles by monocyte and macrophage cell lines. ACS Nano.

[65] Baimanov D. (2020). Immunological Responses Induced by Blood Protein Coronas on Two-Dimensional
MoS(2) Nanosheets. ACS Nano.

[66] Bashiri G. (2023). Nanoparticle protein
corona: from structure and function to therapeutic
targeting. Lab Chip.

[67] Baumann C., Zerbe O. (2024). The role of leucine
and isoleucine in tuning the hydropathy of class
A GPCRs. Proteins.

[68] Deber C. M., Stone T. A. (2019). Relative role(s)
of leucine versus isoleucine in the
folding of membrane proteins. Peptide Sci..

[69] Brohee S., van Helden J. (2006). Evaluation
of clustering algorithms for protein-protein
interaction networks. BMC Bioinf..

[70] Baxter E. W., Graham A. E., Re N. A., Carr I. M., Robinson J. I., Mackie S. L., Morgan A. W. (2020). Standardized
protocols for differentiation
of THP-1 cells to macrophages with distinct M­(IFNγ+LPS), M­(IL-4)
and M­(IL-10) phenotypes. J. Immunol. Methods.

[71] Perez-Riverol Y. (2022). The PRIDE database resources in 2022: a hub
for mass spectrometry-based
proteomics evidences. Nucleic Acids Res..

