# Supplementary material for: Isomeric Differences in Nanoparticle’s Surface Chemistry Alter Macrophage Interactions In Vitro Due to Protein Corona
Source: ACS Omega. 2025 Sep 11;10(37):42346–59. doi: 10.1021/acsomega.5c03123 (PMC12461424; doi:10.1021/acsomega.5c03123)

Isomeric Differences in Nanoparticle's Surface Chemistry Alters Macrophage Interactions In Vitro Due to Protein Corona

Sridevi B. Conjeevaram, Amulya Kadaba, Isaac M. Adjei*

Department of Biomedical Engineering, Texas A&M University, College Station, TX 77843

*Corresponding author:

Sridevi B. Conjeevaram

Department of Biomedical Engineering

Texas A&M University

College Station, TX 77843

Ph: 3528715518

Email: sconjeevaram@tamu.edu

Figure S1: Intensity, Number and volume distribution of A) Leu AUNP PC and B) Iso Leu AuNP PC presented in triplicates.

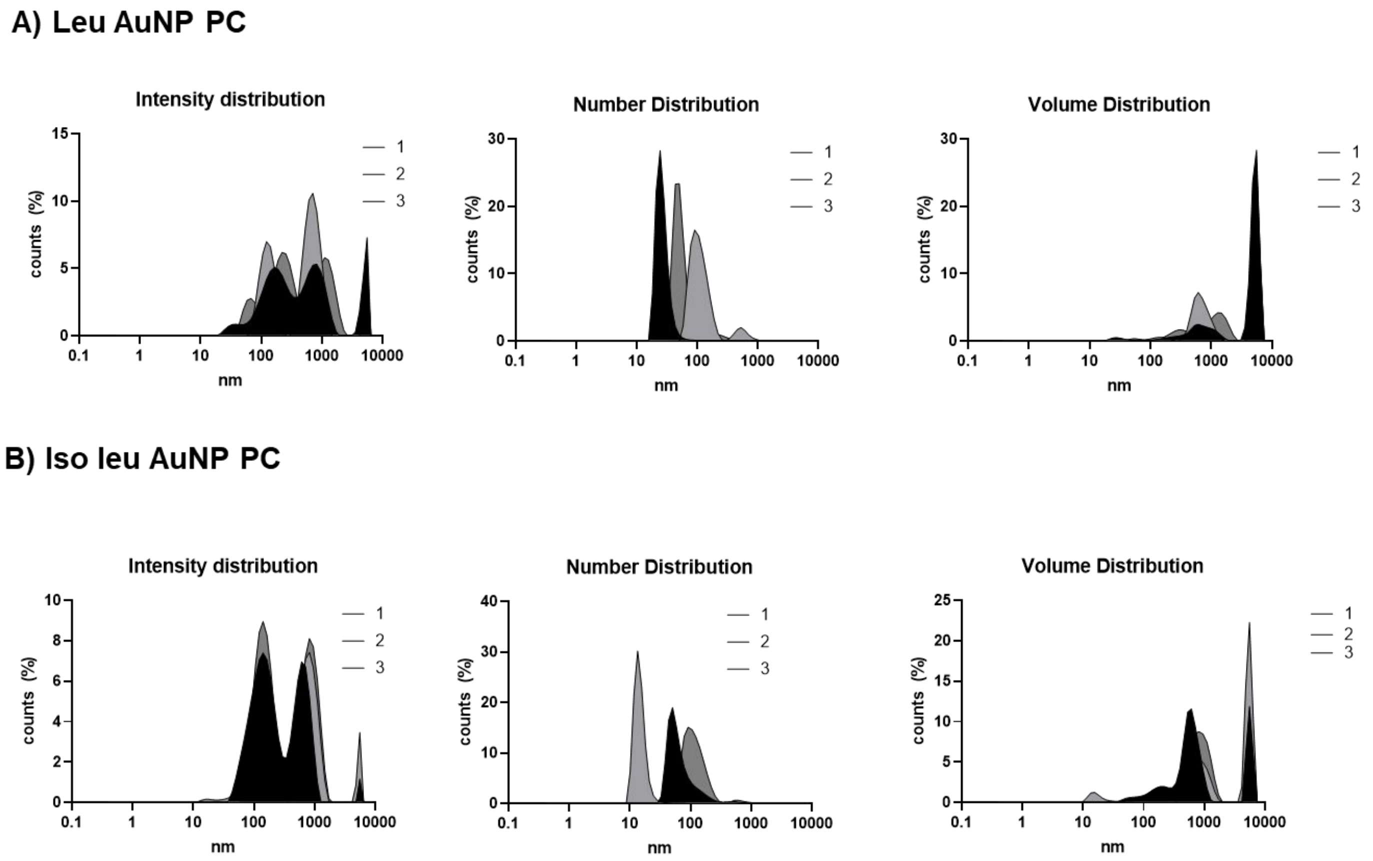

Figure S2: Comparison of Number and Intensity distribution before and after protein corona (PC) formation on A) Leu AuNP and B) Iso Leu AuNP. Average of three runs was calculated and used in the reporting here.

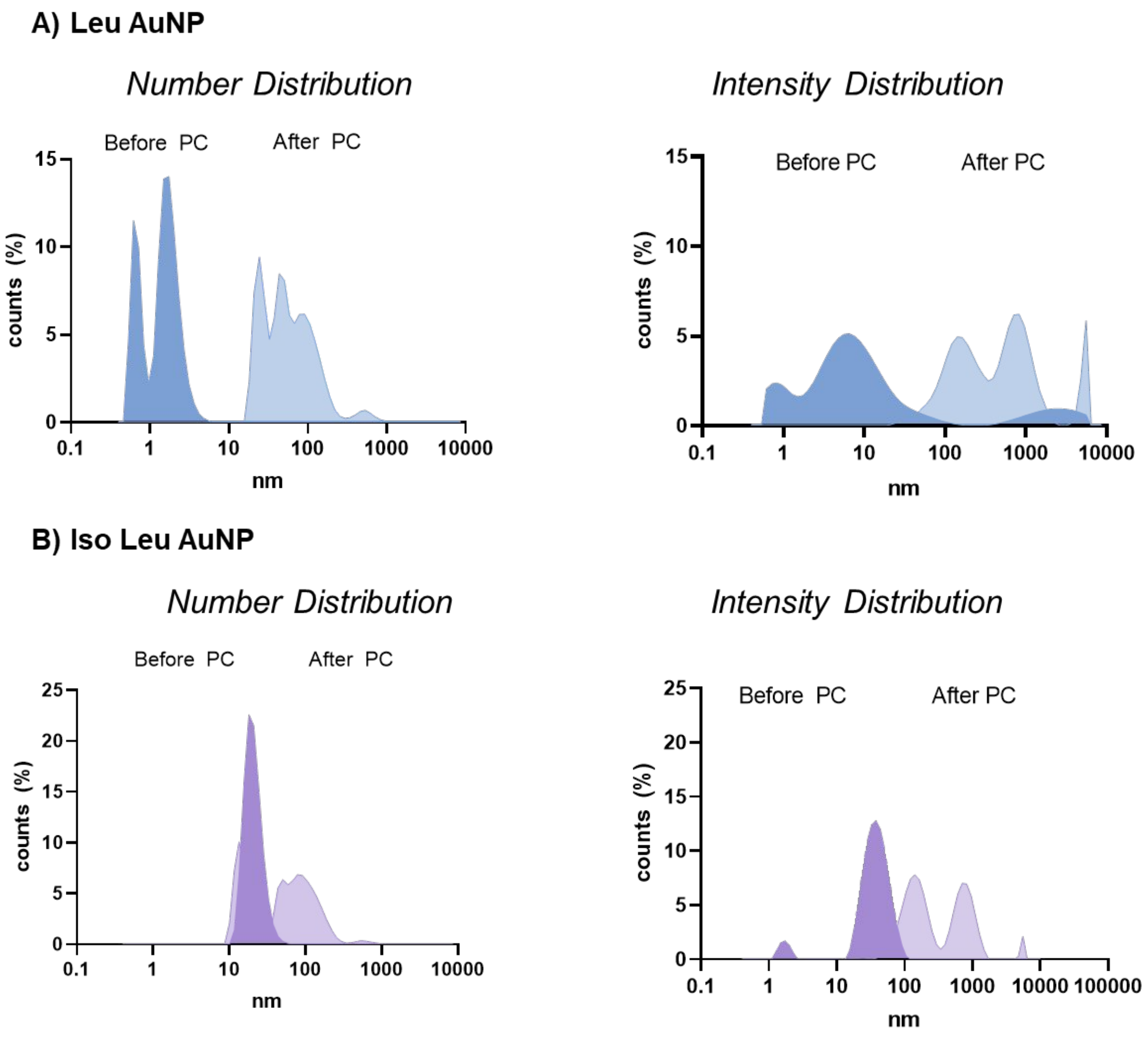

Figure S3: Zeta potential kinetics of NP-PC over time

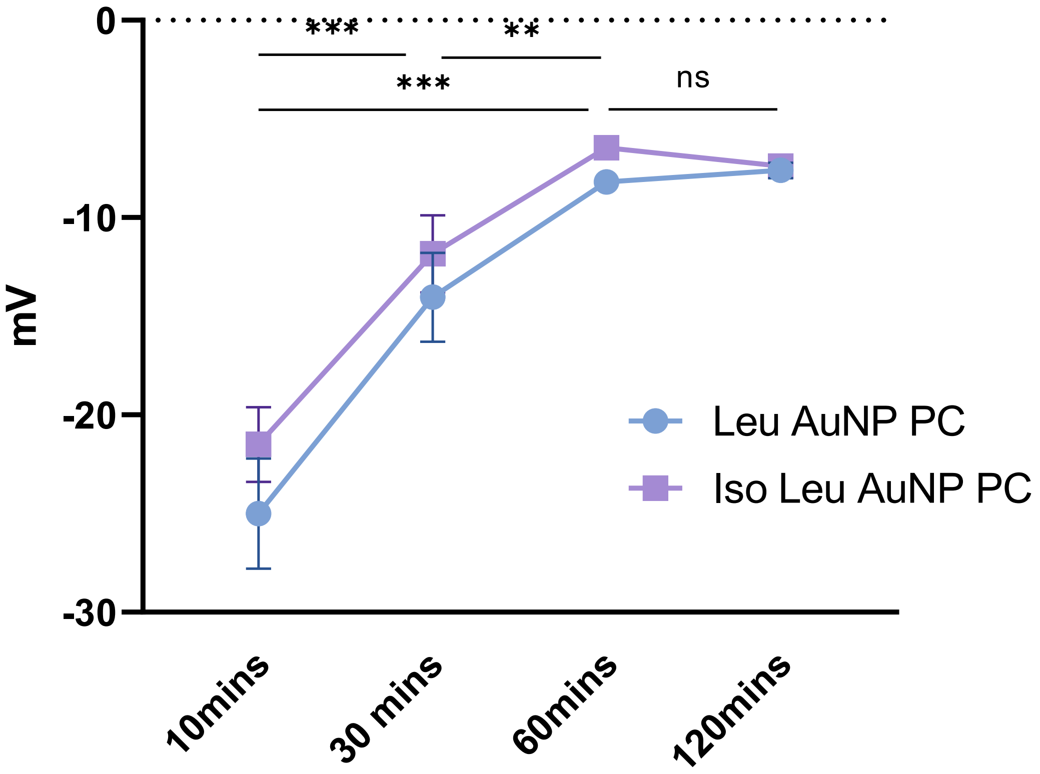

Figure S4: dTHP Macrophages only without NP treatment

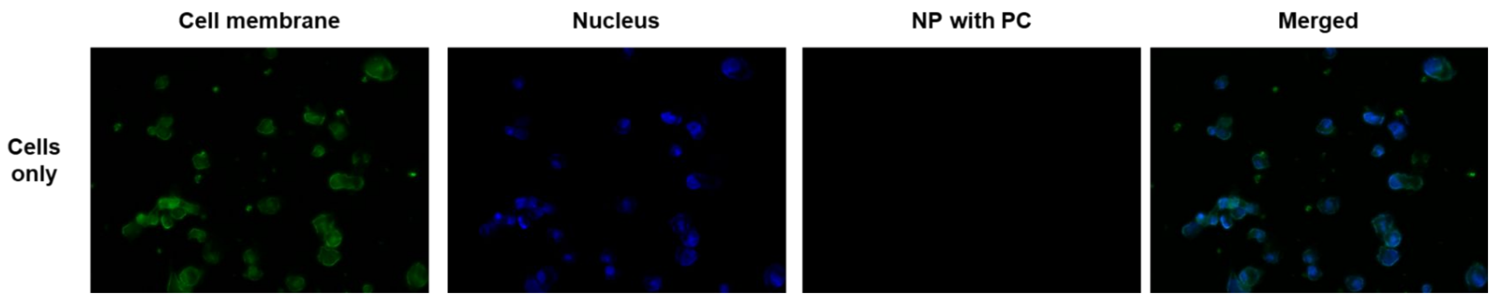

Supplement: Supplementary file 2 [file ao5c03123_si_002.pdf]
